# Noninvasive MRA-derived fractional flow for intracranial stenosis: methodological evaluation and hemodynamic insights

**DOI:** 10.3389/fneur.2026.1808722

**Published:** 2026-06-29

**Authors:** Xiaohui Wang, Haojing Zhu, Zhihua Du, Rongju Zhang, Yang Bian, Xinfeng Liu, Bin Lv, Rui Zhang, Jinhao Lyu, Xu Guan, Ziwei Guo, Xiangyu Cao, Jiawen Zhu, Rong Zou, Jianping Xiang, Shengyuan Yu, Jun Wang

**Affiliations:** 1 Department of Neurology, The First Medical Center of PLA General Hospital, Beijing, China; 2 Department of Radiology, Chinese PLA General Hospital, Beijing, China; 3 Department of Hyperbaric Oxygen, The First Medical Center of PLA General Hospital, Beijing, China; 4 ArteryFlow Technology Co., Ltd., Hangzhou, China

**Keywords:** fractional flow, hemodynamic impairment, intracranial atherosclerotic stenosis, magnetic resonance angiography, noninvasive assessment

## Abstract

**Objective:**

Current fractional flow (FF) approaches in quantifying the hemodynamic severity of intracranial atherosclerotic stenosis (ICAS) are invasive or require angiography. This study primarily evaluated a noninvasive FF method derived from magnetic resonance angiography (MRA-FF) against established reference standards, with an exploratory assessment of its ability to reflect hemodynamically relevant impairment.

**Methods:**

In this retrospective study of 50 patients with ICAS, MRA-FF was computed from 3D vascular models using patient-specific flow boundary conditions. Agreement with invasive pressure-wire-derived FF (PW-FF, *n* = 18) and angiography-based FF (DSA-FF, *n* = 50) was assessed using intraclass correlation coefficients (ICC). Equivalence to DSA-FF was evaluated using the two one-sided tests procedure (TOST; *δ* = ±0.05). An exploratory analysis examined the ability of MRA-FF to discriminate hypoperfusion using receiver operating characteristic (ROC) analysis in the subset of patients with available perfusion imaging (*n* = 38).

**Results:**

MRA-FF showed moderate-to-good agreement with PW-FF (ICC = 0.743; 95% CI, 0.437–0.895) and good agreement with DSA-FF (ICC = 0.784; 95% CI, 0.649–0.871). Equivalence to DSA-FF was confirmed by TOST (90% CI of difference, −0.016 to 0.035; *p* < 0.01). In the perfusion subset, MRA-FF was lower in patients with hypoperfusion than in those without (*p* = 0.022). The area under the ROC curve for discriminating hypoperfusion was 0.723 (95% CI, 0.54–0.90). Within this dataset, the exploratory optimal cutoff value was 0.855.

**Conclusion:**

MRA-FF demonstrates good agreement with invasive and angiographic reference methods and shows the ability to discriminate hypoperfusion in an exploratory analysis. These findings support the feasibility and potential clinical utility of a fully noninvasive FF approach for functional assessment and hemodynamic characterization, although further external validation is required.

## 1 Introduction

Fractional flow (FF), defined as the ratio of distal to proximal pressure across a stenosis, provides a physiologic assessment of lesion-specific hemodynamic severity (1). It is a cornerstone of functional assessment in coronary artery disease (2–4). With increasing insight into cerebrovascular pathophysiology, the concept of FF has been extended to intracranial atherosclerotic stenosis (ICAS) (5–7). Prior studies show that patients with similar degrees of angiographic stenosis may exhibit markedly different downstream perfusion and ischemic risk (8–11). This discrepancy highlights the limitations of anatomy-based management and underscores the need for lesion-specific functional metrics in cerebrovascular practice.

However, translating FF to the intracranial circulation is hampered by practical challenges: current acquisition methods, whether invasive pressure-wire measurements or computational methods derived from digital subtraction angiography (DSA) require arterial access and contrast administration (12, 13). These requirements introduce procedural risks, potential toxicity, and significant costs, which collectively preclude the use of existing FF techniques for large-scale screening, serial monitoring, or long-term follow-up.

Magnetic resonance angiography (MRA) provides a noninvasive alternative. As a routine imaging technique that requires neither radiation nor contrast, MRA offers inherent advantages in safety, accessibility, and reproducibility. Crucially, recent advances in image-based vascular modeling and computational fluid dynamics (CFD) allow FF to be derived directly from standard MRA data (14, 15). This approach enables a lesion-specific functional assessment that is entirely noninvasive.

Nevertheless, the quantitative accuracy of MRA-derived FF (MRA-FF) and its ability to discriminate hemodynamic abnormalities in ICAS patients remain to be systematically validated. To address this gap, the present study was designed to primarily evaluate the agreement of an MRA-FF calculation method with established reference techniques. In addition, an exploratory analysis was performed to examine the relationship between MRA-FF and perfusion-defined abnormalities. Together, these analyses provide an initial assessment of the feasibility and translational relevance of a fully noninvasive tool for functional evaluation of ICAS.

## 2 Materials and methods

### 2.1 Study population

This study was conducted in accordance with the Declaration of Helsinki. We retrospectively reviewed the clinical and imaging data of patients admitted to the Chinese PLA General Hospital between January 2021 and June 2024 for ischemic stroke or transient ischemic attack caused by ICAS. The final study cohort was defined based on the following inclusion and exclusion criteria. Inclusion criteria were: (1) age 18–80 years; (2) completion of magnetic resonance angiography (MRA) within 1 week prior to digital subtraction angiography (DSA); and (3) stenotic lesions responsible for the ischemic event located in the intracranial internal carotid artery, middle cerebral artery, or from the V4 segment of the vertebral artery to the basilar artery apex. Exclusion criteria were: (1) poor-quality DSA or MRA that precluded subsequent analyses; (2) incomplete or unavailable transcranial Doppler (TCD) data for the target vessel; (3) ischemic events attributable to non-atherosclerotic causes, such as moyamoya disease, vasculitis, arterial dissection, or cardioembolism; and (4) previous revascularization surgery or endovascular intervention targeting the responsible vessel. Subset analyses were performed based on data availability, as detailed in the corresponding sections of the Results.

The study protocol was approved by the Ethics Committee of the Chinese PLA General Hospital (S2021-177-01). The ethics committee waived the requirement for informed consent due to the retrospective, anonymized nature of the study.

### 2.2 Imaging data acquisition

#### 2.2.1 DSA

All DSA examinations were performed at our institution using a biplane angiography system (Allura Xper FD20, Philips Healthcare, the Netherlands). Following transfemoral access, the diagnostic catheter was selectively advanced to the proximal segment of the target intracranial artery. Iodinated contrast medium was power-injected at a rate of 3–5 mL/s (total dose, 5–7 mL) to achieve optimal vessel opacification. Image acquisition was performed at a frame rate of 4 frames per second.

#### 2.2.2 MRA

MRA data were acquired for three-dimensional vascular reconstruction and CFD-based hemodynamic analysis. All examinations were performed on a 3.0 T MR scanner (Discovery MR750, GE Healthcare, the United States) with a 32-channel head coil. Three-dimensional time-of-flight (3D TOF) MRA was used for analysis. The key acquisition parameters were as follows: repetition time (TR), 34 ms; echo time (TE), 3.1 ms; field of view (FOV), 24 cm; matrix, 512 × 128; slice thickness, 1.0 mm; slice overlap, 0.5 mm; total number of slices, 152; and number of excitations (NEX), 1. Source images were archived in Digital Imaging and Communications in Medicine (DICOM) format.

#### 2.2.3 TCD

TCD data were used to provide patient-specific inflow velocity conditions for CFD-based hemodynamic analysis. TCD examinations were performed within 48 h of admission using a dedicated system (Doppler Box, DWL, Germany) by two certified neurosonographers. The ipsilateral parent artery proximal to the target stenosis was insonated to obtain the time-averaged mean velocity. The final inflow velocity for subsequent simulations was calculated as the mean of three consecutive measurements.

#### 2.2.4 Preoperative cerebral perfusion imaging

Preoperative cerebral perfusion imaging was postprocessed on a dedicated workstation (AW Workstation 4.3, GE Healthcare, the United States). Perfusion images were acquired with post-labeling delays of 1.5 s and 2.5 s to account for arterial transit delay. Quantitative cerebral blood flow (CBF) maps were generated on a dedicated workstation. Hypoperfusion was defined as a regional reduction in CBF compared with the contralateral territory and assessed separately at each PLD. Delayed flow was identified by a reduction in the extent of hypoperfusion from 1.5 s to 2.5 s. Collateral flow was classified as favorable if delayed flow involved more than 50% of the hypoperfused region (16–18).

### 2.3 FF calculation and workflow

#### 2.3.1 Pressure-wire-derived FF (PW-FF) measurement

Pressure-wire measurements were performed under general anesthesia as part of a standard neurointerventional procedure. An intermediate or guiding catheter was positioned at the proximal segment of the target vessel. A 0.014-inch pressure wire (C12008; Abbott St. Jude Medical, the United States) was then advanced across the stenosis through a microcatheter and placed at least 2–3 cm distally. Resting pressures were recorded simultaneously proximal (P_a_) and distal (P_d_) to the stenosis. PW-FF was calculated as the ratio of P_d_ to P_a._

#### 2.3.2 DSA-based FF (DSA-FF) calculation

DSA-based FF was computed using AccuICAD (version 1.0; ArteryFlow Technology, China). The software’s core algorithm and diagnostic performance have been validated in previous studies (19). Briefly, the software automatically selected optimal angiographic frames and extracted the centerline and lumen contours of the target vessel. Patient-specific blood flow velocity was then obtained using the thrombolysis in myocardial infarction (TIMI) frame-counting method (20). The velocity data were used to solve the governing fluid dynamics equations, generating the DSA-FF value for the stenotic lesion.

#### 2.3.3 MRA-FF calculation

Three-dimensional vascular models were reconstructed from intracranial MRA DICOM data using MIMICS (version 20.0; Materialise, Belgium), with collateral branches <50% of the parent vessel diameter excluded. For each model, a polyhedral volumetric mesh with prismatic layers near the vessel wall was generated. The maximum cell size was set to 0.15 mm, resulting in approximately 1–2 million cells per model. The inlet boundary condition was prescribed using the patient-specific, time-averaged velocity obtained from TCD measurements (21). Patient-specific arterial blood pressure measured proximal to the stenosis during the peri-procedural period was incorporated as the reference pressure for FF calculation. Outlets were governed by split outlet boundary conditions, where the outflow rate was proportional to the cube of the corresponding outlet diameter (22, 23).

**Figure 1:**
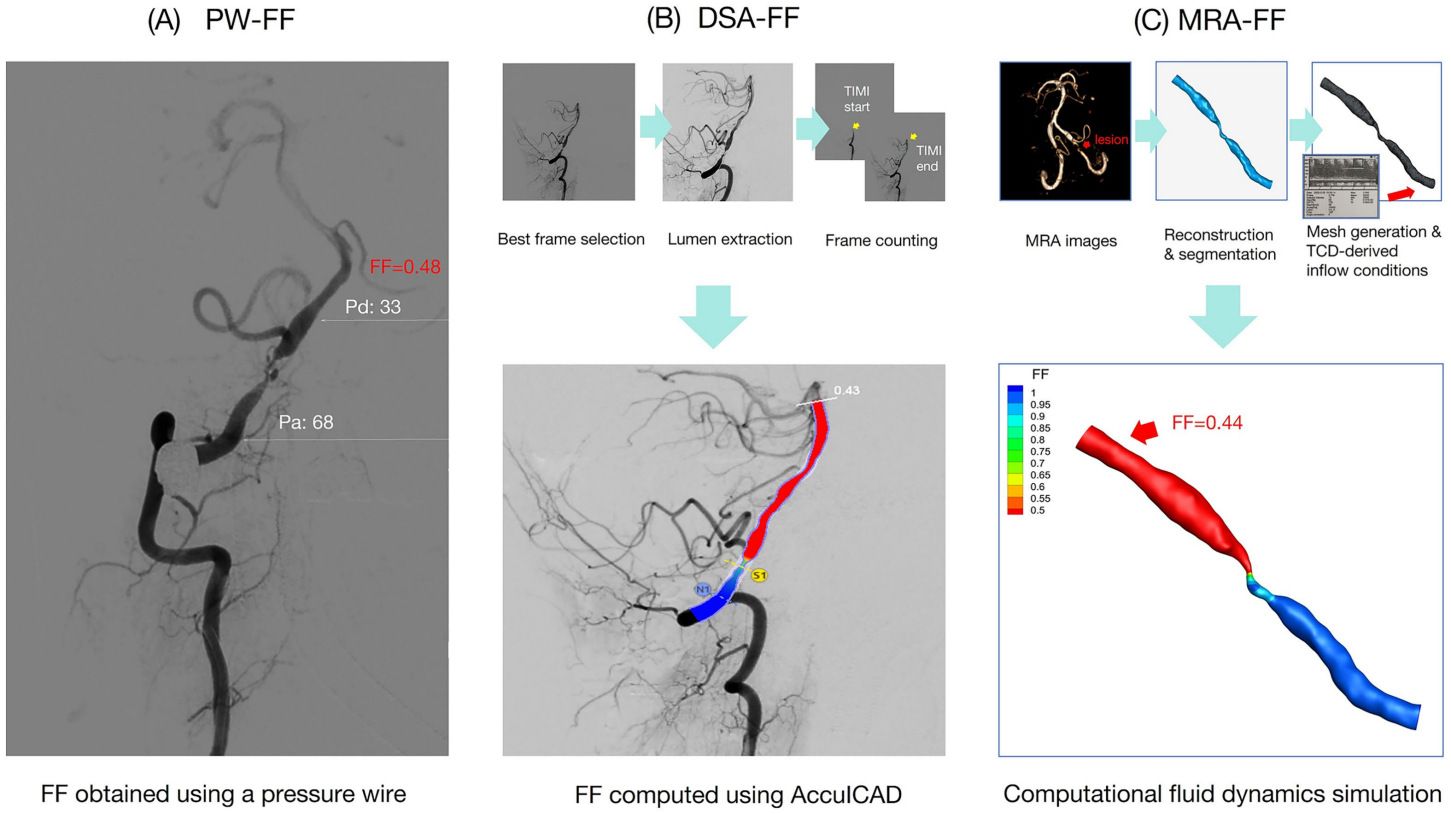
Representative workflows for intracranial fractional flow (FF) acquisition. Representative case: a 60-year-old man with symptomatic right vertebral artery stenosis. **(A)** Pressure-wire-derived fractional flow (PW-FF). **(B)** Digital subtraction angiography-derived fractional flow (DSA-FF). **(C)** Magnetic resonance angiography-derived fractional flow (MRA-FF). Representative processing steps are illustrated within each workflow.

Hemodynamic simulations were conducted using the finite volume method in STAR-CCM + (version 20.02; Siemens Digital Industries Software, the United States). Blood was modeled as an incompressible Newtonian fluid (density, 1,056 kg/m^3^; dynamic viscosity, 0.0035 Pa·s), and vessel walls were assumed rigid with no-slip boundary conditions (24, 25). Steady-state simulations were performed under mean-flow conditions, neglecting pulsatile effects. The SIMPLE algorithm was employed for pressure–velocity coupling with second-order spatial discretization. Convergence was defined as normalized residuals for both the continuity and momentum equations falling below 10^−5^. MRA-FF was defined as the computed ratio of distal to proximal pressure across the stenosis (P_distal_/P_proximal_). Representative workflows for PW-FF, DSA-FF, and MRA-FF acquisition and analysis are illustrated in Figure 1.

### 2.4 Statistical analysis

Statistical analyses were conducted using R (version 4.5.1; R Foundation for Statistical Computing, Austria) within RStudio (version 2025.09.1; Posit PBC, the United States). Continuous data were summarized as mean ± standard deviation or median (interquartile range) based on their distribution, assessed via the Shapiro–Wilk test. Accordingly, group differences were evaluated using the independent-samples t-test or the Mann–Whitney U test (with Cliff’s delta effect sizes calculated) (26); categorical variables were compared with Fisher’s exact test. Agreement was quantified with the intraclass correlation coefficient (ICC) (27) and visualized with Bland–Altman plots. Equivalence between paired measurements was tested using the two one-sided tests (TOST) procedure, with a prespecified equivalence margin (*δ*) of ±0.05 (28, 29). Diagnostic accuracy was quantified using the area under the receiver operating characteristic (ROC) curve (AUC). The optimal cutoff was identified by maximizing the Youden index. All tests were two-sided, and a *p*-value <0.05 was considered statistically significant.

## 3 Results

### 3.1 Baseline characteristics

**Figure 2:**
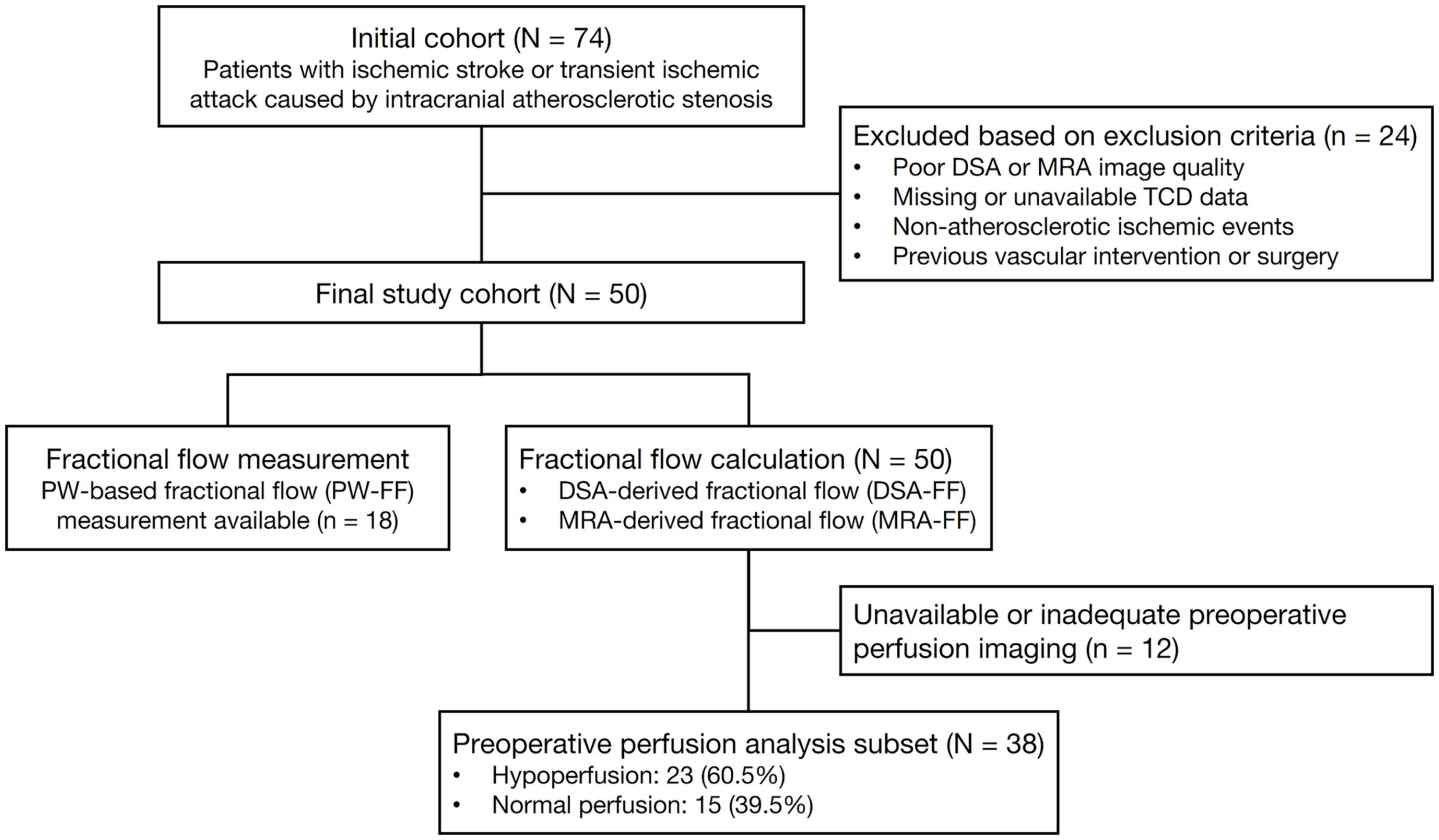
Study flowchart.

Of 74 patients with ICAS who were screened, 24 were excluded according to the predefined criteria, resulting in a final cohort of 50 patients (Figure 2). The baseline characteristics are summarized in Table 1. MRA-FF and DSA-FF were computed for all 50 patients. PW-FF was additionally available in a subset of 18 patients. Thirty-eight patients (76%) had preoperative perfusion imaging data of sufficient quality for analysis. Perfusion data were unavailable in the remaining 12 patients due to the absence of perfusion imaging (*n* = 4) and non-diagnostic image quality (*n* = 8).

**Table 1:** Baseline characteristics of the study cohort (*N* = 50).

Characteristic	Value
Demographics	
Age, y	59.7 ± 8.7
Male sex, n (%)	40 (80)
Body mass index, kg/m^2^	25.5 ± 2.6
Vascular risk factors, *n* (%)	
Hypertension	34 (68)
Diabetes mellitus	17 (34)
Hyperlipidemia	14 (28)
Current smoking	26 (52)
Alcohol consumption	22 (44)
Stenosis location, *n* (%)	
Internal carotid artery	11 (22)
Middle cerebral artery	19 (38)
Vertebral artery	7 (14)
Basilar artery	13 (26)

### 3.2 Agreement and equivalence of MRA-FF measurements

#### 3.2.1 Comparison of MRA-FF with invasive PW-FF

**Figure 3:**
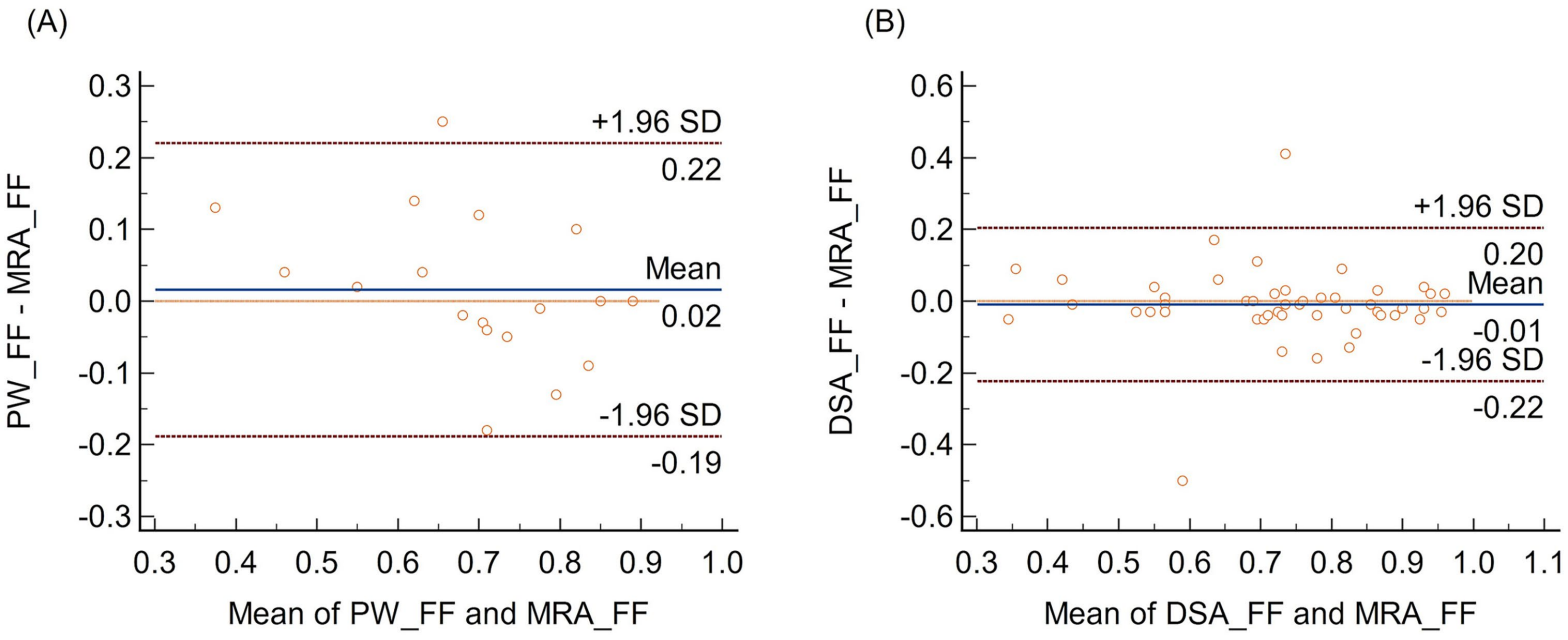
Bland–Altman plots. **(A)** Comparison between MRA-FF and PW-FF in 18 patients. **(B)** Comparison between MRA-FF and DSA-FF in 50 patients.

In the 18-patient subset with concurrent pressure-wire measurements, mean values for MRA-FF and PW-FF were 0.69 ± 0.16 and 0.70 ± 0.12, respectively. The two measures showed moderate-to-good agreement, with an ICC of 0.743 (95% CI, 0.437–0.895). Bland–Altman analysis demonstrated a mean difference of 0.016 (95% CI, −0.036–0.068), with 95% LoA ranging from −0.189 to 0.221. No significant proportional bias was detected on regression analysis (slope *p* = 0.10; Figure 3A).

#### 3.2.2 Comparison of MRA-FF with validated DSA-FF

In the full cohort of 50 patients, mean values for MRA-FF and DSA-FF were 0.74 ± 0.17 and 0.73 ± 0.16, respectively. Good agreement was observed between the two measures, with an ICC of 0.784 (95% CI, 0.649–0.871). Bland–Altman analysis revealed a mean difference of −0.010 (95% CI, −0.041–0.021), with 95% LoA ranging from −0.223 to 0.204. No significant proportional bias was detected (slope *p* = 0.953; Figure 3B). The TOST procedure demonstrated statistical equivalence between the two methods, as the 90% CI of the difference (−0.016–0.035) lay entirely within the prespecified *δ* (*p* < 0.01).

### 3.3 Discriminative performance of MRA-FF for preoperative hypoperfusion

The diagnostic accuracy of MRA-FF was assessed in the 38-patient subset with preoperative perfusion imaging of sufficient quality, which showed no significant differences in baseline characteristics compared to the full cohort (see Supplementary Table 1).

**Figure 4:**
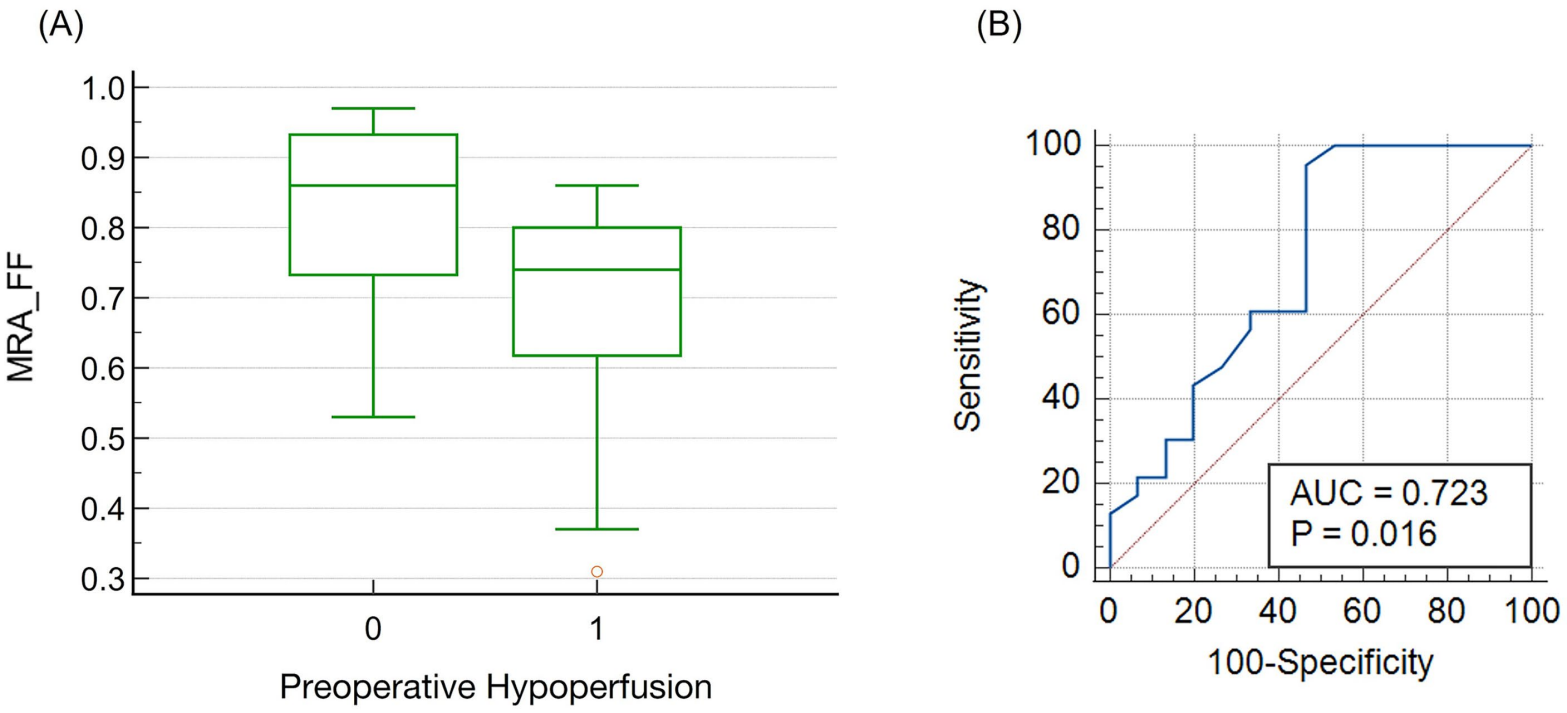
Association between MRA-FF and preoperative hypoperfusion. **(A)** Box-and-whisker plots of MRA-FF values stratified by perfusion status. **(B)** ROC curve of MRA-FF for discriminating hypoperfusion.

Of the 38 patients, 23 (60.5%) exhibited hypoperfusion in the target territory. The median MRA-FF was significantly lower compared with the normal perfusion group [0.74 (IQR 0.63–0.80) vs. 0.86 (IQR 0.74–0.93); Mann–Whitney U = 249.5; *p* = 0.022], with an effect size of Cliff’s delta = −0.45 (95% CI, −0.71 to −0.06). The distributions of MRA-FF values are depicted in Figure 4A.

The ROC curve for MRA-FF in discriminating hypoperfusion had an AUC of 0.723 (95% CI, 0.54–0.90), indicating moderate discriminatory power (Figure 4B). The exploratory optimal FF cutoff derived from this dataset was 0.855, corresponding to a sensitivity of 95.7% (95% CI, 86.9–100%) and specificity of 53.3% (95% CI, 26.7–80.0%). These performance estimates should be interpreted cautiously given the limited sample size and lack of external validation.

## 4 Discussion

The present study demonstrates that a physiologically meaningful fractional flow (MRA-FF) can be computed noninvasively by applying patient-specific flow conditions to vascular models derived from routine MRA data. By bypassing intravascular contrast administration and catheterization, this approach offers advantages in safety, reproducibility, and longitudinal follow-up. It is therefore well suited to clinical scenarios requiring repeated evaluation of lesion-related functional status, with the potential to reduce procedural burden in selected contexts.

In the current study, DSA-FF analysis typically required approximately 10 min per case, whereas MRA-FF analysis required approximately 15 min, including vascular reconstruction, mesh generation, and CFD computation. These characteristics suggest that the different FF approaches may serve complementary clinical roles. DSA-based approaches may be advantageous for rapid angiography-based functional assessment, whereas the fully noninvasive nature of MRA-FF may facilitate longitudinal monitoring, screening, and evaluation in patients who are unsuitable for invasive assessment or repeated contrast exposure.

Importantly, the intended role of MRA-FF is not to replace established invasive measurements, but to complement the current anatomy-centered diagnostic framework by providing a fully noninvasive functional metric that can be readily integrated into routine imaging workflows. Such an approach may expand the availability of hemodynamic assessment in clinical settings where invasive evaluation is impractical or less desirable, while offering supplementary information for functional characterization.

Previous studies in both coronary and cerebrovascular systems report that noninvasive computational approaches can capture aspects of the functional impact of stenotic lesions, typically showing moderate to good agreement with pressure-wire measurements (19, 30–33). The ICC and Bland–Altman agreement analyses in the present cerebrovascular cohort were comparable to those reported in prior studies, suggesting stable quantitative performance even under the complex anatomic configurations and hemodynamic conditions characteristic of cerebral arteries. In parallel, equivalence testing against DSA-FF across the full cohort demonstrated that MRA-FF met the prespecified criteria, further supporting an acceptable level of methodological agreement with previously validated approaches. The equivalence margin (*δ* = ±0.05) was defined based on clinically acceptable numerical differences for this metric, within which variations in FF are generally considered unlikely to meaningfully affect functional grading or clinical decision-making (29). In aggregate, these findings provide a statistical foundation for considering MRA-FF as a viable, fully noninvasive functional metric for clinical research and practice.

In the preprocedural perfusion imaging subset, MRA-FF values were significantly lower in those with hypoperfusion. ROC analysis demonstrated a moderate ability of MRA-FF to discriminate hypoperfusion (AUC = 0.723), characterized by high sensitivity and moderate specificity. The relatively high negative predictive value of MRA-FF suggests that, within this cohort or in comparable clinical settings, a negative result may help rule out hemodynamically significant stenosis. It is important to note, however, that perfusion and FF represent distinct functional constructs. Fractional flow characterizes the local hemodynamic constraint imposed by a stenosis under specific flow conditions, whereas perfusion imaging reflects the integrated tissue-level blood supply, which is influenced by collateral circulation and autoregulatory mechanisms. As a result, an abnormal FF does not necessarily indicate abnormal perfusion, and vice versa. Therefore, the ROC analysis and corresponding thresholds reported here should be interpreted as exploratory and dataset-specific, illustrating associations between MRA-FF and clinical functional phenotypes rather than establishing definitive diagnostic cutoffs. External validation in larger independent cohorts is still required before broader clinical application. Collectively, MRA-FF and perfusion imaging provide complementary insights: the former assesses lesion-specific upstream hemodynamic severity, and the latter captures downstream tissue-level consequences. These perspectives offer a more nuanced functional characterization of symptomatic ICAS, supporting their potential utility in clinical research and patient-level hemodynamic assessment (5).

The hemodynamic model employed in this study relies on commonly accepted simplifying assumptions that are standard in computational modeling of cerebral arteries. While these assumptions omit certain physiological details, the present framework still incorporates several patient-specific components, including individualized vascular geometry, TCD-derived inflow conditions, and patient-specific blood pressure measurements. In addition, FF fundamentally represents the pressure ratio across a stenosis rather than absolute pressures, rendering it relatively insensitive to detailed flow properties. Previous studies also have shown that vessel geometry and boundary conditions generally exert a greater influence than the precise flow properties (34, 35). Likewise, the TCD-derived patient-specific flow scaling is used to calibrate relative pressures rather than to precisely replicate instantaneous velocity fields, making its effect on FF generally secondary (33). The application of Murray’s law to distribute flow similarly reflects a widely used empirical strategy in cerebral artery modeling, aimed at preserving relative changes within a vascular tree rather than explicitly simulating microcirculatory regulation. Overall, these well-established approximations allow the model to capture the dominant hemodynamic impact of a stenosis while maintaining computational tractability.

This study has limitations. The small sample size of the pressure-wire validation subset (*n* = 18) limits the precision of agreement estimates with the physiological reference; therefore, the corresponding analysis should be considered exploratory. Accordingly, equivalence testing was not performed within the PW-FF subset. Instead, the primary methodological validation of MRA-FF in the present study was based on agreement and equivalence analyses with the larger DSA-FF cohort (*n* = 50). In the perfusion imaging subset, the limited number of outcome events (*n* = 23) resulted in a stepwise appearance of the ROC curve, indicating that the derived cutoff and associated performance metrics are dataset-specific and should be interpreted cautiously pending external validation.

## 5 Conclusion

The present study demonstrates the feasibility of a fully noninvasive MRA-based method for functional assessment of ICAS. MRA-FF showed good agreement with invasive pressure-wire measurements and a validated angiographic computational method and was associated with perfusion abnormalities in this cohort. These findings support the potential role of MRA-FF as a supplemental, noninvasive reference for functional characterization and risk stratification in ICAS, although further external validation is required before broader clinical application.

## Data Availability

The raw data supporting the conclusions of this article will be made available by the authors, without undue reservation.
