# Supplementary material for: Noninvasive MRA-derived fractional flow for intracranial stenosis: methodological evaluation and hemodynamic insights
Source: Front Neurol. 2026 Jun 29;17:1808722. doi: 10.3389/fneur.2026.1808722 (PMC13357202; doi:10.3389/fneur.2026.1808722)
Supplement: Supplementary file 1 [file Table_1.DOCX]

Supplementary Material

**Supplementary Table 1.** Baseline characteristics of patients with and without preoperative perfusion data

| **Characteristic** | **With perfusion data (n = 38)** | | **Without perfusion data (n = 38)** | **P value** | |
| --- | --- | --- | --- | --- | --- |
| Age, y | 58.55 ± 7.90 | 63.42 ± 10.30 | | | 0.153 |
| Male sex, n (%) | 30 (78.9%) | 10 (83.3%) | | | 1.000 |
| Body mass index, kg/m² | 25.64 ± 2.75 | 25.03 ± 2.05 | | | 0.421 |
| Hypertension, n (%) | 25 (65.8%) | 9 (75.0%) | | | 0.728 |
| Diabetes mellitus, n (%) | 12 (31.6%) | 5 (41.7%) | | | 0.728 |
| Hyperlipidemia, n (%) | 10 (26.3%) | 4 (33.3%) | | | 0.718 |
| Preoperative mRS score | 0.5 (0–1) | 1 (0–1) | | | 0.406 |
| DSA-derived fractional flow | 0.72 ± 0.16 | 0.74 ± 0.18 | | | 0.836 |
| Anterior circulation stenosis, n (%) | 23 (60.5%) | 7 (58.3%) | | | 1.000 |
